# A comparative study on the clinical outcomes of local flap transfer and free flap transplantation for the repair of lower limb bone exposure wounds

**DOI:** 10.3389/fsurg.2025.1682145

**Published:** 2025-11-17

**Authors:** Xianglong Li, Xiangbin Zhao, Kang Guo, Xueyang Li

**Affiliations:** 1 Department of Plastic and Burn Reconstruction Surgery, Huai'an Hospital affiliated to Xuzhou Medical University, Huaian, Jiangsu, China; 2 Department of Burn Plastic Surgery, The Second Affiliated Hospital of Xuzhou Medical University, Xuzhou, Jiangsu, China; 3 Department of Burn Plastic Surgery, Shangqiu First People’s Hospital, Shangqiu, Henan, China; 4 Department of Plastic Surgery, The Affiliated Hospital of Xuzhou Medical University, Xuzhou, Jiangsu, China

**Keywords:** local flap transfer, free flap transplantation, lower limb bone exposure wounds, complications, quality of life

## Abstract

**Objective:**

To compare and analyze the clinical outcomes of local flap transfer and free flap transplantation for the repair of lower limb bone exposure wounds.

**Methods:**

A retrospective study was conducted on 90 patients with lower limb bone exposure wounds admitted to our hospital from December 2022 to January 2024. Patients were divided into two groups based on the type of flap repair: the local flap group (*n* = 50) and the free flap group (*n* = 40). The local flap group underwent local flap transfer repair, while the free flap group underwent free flap transplantation repair. The two groups were compared in terms of surgical duration, intraoperative blood loss, hospital stay, complication incidence, and 1-year postoperative aesthetic satisfaction, functional recovery rate, and quality of life (SF-36 questionnaire).

**Results:**

Compared with the local flap group, the free flap group had longer surgical duration, greater intraoperative blood loss, and shorter hospital stay, with statistically significant differences (*P* < 0.05). The incidence of complications in the local flap group (10.00%) was significantly lower than that in the free flap group (30.00%) (*P* < 0.05). One year postoperatively, the aesthetic satisfaction rate in the free flap group (92.50%) was slightly higher than that in the local flap group (82.00%), but the difference was not statistically significant (*P* = 0.145). The rate of excellent functional recovery in the free flap group (87.50%) was significantly higher than that in the local flap group (70.00%, *P* = 0.047), and the scores for eight dimensions of the SF-36 questionnaire, including physical pain and physical function, were significantly higher (all *P* < 0.01).

**Conclusion:**

Local flap transfer has the advantages of less surgical trauma and fewer complications; although free flap transplantation is more complex in operation and has a higher risk of complications, it has more advantages in functional recovery and improvement of quality of life.

## Introduction

Lower limb bone exposure wounds are common clinical trauma conditions that can be caused by various factors such as open fractures, post-traumatic soft tissue necrosis, and infections (1, 2). These wounds not only affect the patient's appearance but can also lead to severe complications such as osteomyelitis and nonunion, significantly impacting limb function and quality of life (3). Timely and effective repair is crucial for restoring limb integrity, preventing infection, and promoting functional recovery.

Flap transplantation is a commonly used method for repairing lower limb bone exposure wounds, with local flap transfer and free flap transplantation being widely applied. Local flap transfer utilizes skin and subcutaneous tissue adjacent to the bone exposure site. By designing an appropriate flap shape and pedicle, the flap is transferred to cover the wound (4, 5). This technique supports fracture healing, has low complication and infection rates, and demonstrates the advantages of being suitable for certain cases of lower limb bone exposure with higher survival rates (6, 7). Free flap transplantation, on the other hand, involves harvesting a flap with a vascular pedicle from another part of the body (such as the abdomen or lateral thigh), and using microsurgical techniques to anastomose the flap's vessels with those in the recipient area (8). This method can provide larger flaps of varying textures suitable for complex wound repairs; however, it involves more complex surgical procedures, requires high technical expertise, and carries a relatively higher risk of postoperative complications (9, 10).

Currently, there are few comparative studies reporting on the clinical outcomes of patients undergoing local flap transfer vs. free flap transplantation for the repair of lower limb bone exposure wounds. This study aims to compare the clinical outcomes of these two techniques in repairing lower limb bone exposure wounds, providing more valuable reference data for clinical treatment.

## Materials and methods

### Study population

This study was a retrospective analysis. A total of 120 patients with lower limb bone exposure wounds who received treatment at our hospital from December 2022 to January 2024 were selected. After screening according to inclusion and exclusion criteria, 90 patients were selected as the study population. The choice between local flap and free flap reconstruction was primarily based on a comprehensive assessment of individual patient and wound characteristics. Key factors influencing the decision included: the size and depth of the soft tissue defect, the location of the wound (e.g., proximity to joints, weight-bearing areas), the condition of the surrounding tissue and recipient vessels, the presence of concomitant injuries (e.g., tendon or muscle loss), the patient's overall health status and comorbidities (e.g., diabetes, vascular disease), and the surgeon's expertise and institutional resources. Generally, local flaps were preferred for smaller, more superficial defects with well-vascularized adjacent tissue and in patients with higher surgical risk. Free flaps were considered for larger, complex defects, especially those involving exposed vital structures or requiring substantial tissue bulk, and in patients with good physiological reserve and higher demands for functional outcome. Patients were divided into two groups based on the type of flap repair: the local flap group (*n* = 50) and the free flap group (*n* = 40). The local flap group underwent local flap transfer repair, while the free flap group underwent free flap transplantation repair. All patients have signed written informed consent. This study was approved by our hospital's ethics committee and complies with the Declaration of Helsinki. Wound size for all included patients ranged from 1  × 1 cm to 5 × 1 cm as per inclusion criteria. Detailed Gustilo classification for open fractures was not consistently available in the retrospective data.

### Inclusion and exclusion criteria

Inclusion criteria: (1) age between 18 and 65 years; (2) lower limb bone exposure wound size ranging from 1 × 1 cm to 5 × 1 cm; (3) time from injury to hospital presentation within 72 h (extended due to the special nature of bone exposure) (4) no severe dysfunction of vital organs such as the heart, liver, or kidneys.

Exclusion Criteria: (1) patients with uncontrolled severe infections; (2) patients with hematological disorders or coagulation disorders; (3) patients intolerant to flap repair surgery; (4) patients with incomplete clinical data.

### Surgical methods

Both groups of patients underwent routine preoperative examinations (e.g., complete blood count, coagulation function, electrocardiogram, lower limb vascular ultrasound, etc.) and preoperative preparations based on the condition of the wound (e.g., control of underlying diseases, wound cleansing, anti-infection treatment, etc.).

Local Flap Group: Based on the location of the exposed lower limb bone, wound size, shape, and the condition of the surrounding skin tissue, an appropriate local flap (e.g., a sural fasciocutaneous flap, ankle advancement flap, etc.) was designed. The specific types of local flaps used in this cohort included: sural neurofasciocutaneous flap (*n* = 28), reverse superficial sural artery flap (*n* = 12), peroneal artery perforator flap (*n* = 6), and ankle advancement flap (*n* = 4). During surgery, the wound was thoroughly debrided to remove necrotic tissue and foreign bodies, and healthy bone tissue was fully exposed. Then, the flap was harvested according to the design plan. When harvesting the flap, care was taken to preserve its blood supply and avoid damaging vessels and nerves. The harvested flap was transferred to the defect site via rotation, advancement, or other methods and sutured to the area of exposed bone. If the donor site wound could be directly approximated and sutured, it was sutured directly; if not, free skin grafting or other methods were used for repair.

Free flap group: Preoperatively, vascular ultrasound and CT angiography were used to assess the vascular conditions of the donor and recipient sites, and an appropriate free flap was selected (e.g., anterolateral thigh flap, sural flap, etc.). The specific types of free flaps used in this cohort included: anterolateral thigh perforator flap (ALT flap, *n* = 25), thoracodorsal artery perforator flap (TDAP flap, *n* = 8), free superficial circumflex iliac artery perforator flap (SCIP flap, *n* = 5), and free medial sural artery perforator flap (MSAP flap, *n* = 2). During surgery, the wound was first debrided to remove infected and necrotic bone tissue, and then the free flap was harvested under a microscope. When harvesting the flap, the vascular pedicle was carefully dissected to ensure its length and diameter were sufficient for anastomosis. The harvested free flap was then transplanted to the area of bone exposure. Under a microscope, the flap's vascular pedicle was anastomosed with the recipient site's vessels (arteries and veins) to restore blood supply to the flap. After vascular anastomosis, the blood supply of the flap was observed. Once adequate blood supply was confirmed (normal color, temperature, and capillary refill), the flap was sutured to the wound edges. The donor site wound was repaired via direct suturing or free skin grafting as appropriate.

### Clinical data collection

General patient information was collected, including gender, age, BMI, specific location of bone exposure (e.g., proximal, middle, or distal tibia, fibula), and the cause of injury (e.g., open fracture, infection, post-traumatic necrosis, and others).

### Observation indicators and evaluation criteria

1. Surgical-related indicators: The surgical duration, intraoperative blood loss, and hospital stay for both groups of patients were observed and recorded.
2. Complications: Postoperatively, flap necrosis, infection (purulent wound discharge + positive bacterial culture), vascular crisis (arterial spasm/embolism causing flap pallor, venous return obstruction causing flap cyanosis), scar contracture (impairing limb mobility), and osteomyelitis (positive bone culture post-surgery) were observed.
3. Aesthetic satisfaction: One year postoperatively, aesthetic satisfaction in both groups was assessed using a patient-reported outcome measure, categorized into three levels: very satisfied, satisfied, and dissatisfied. Aesthetic satisfaction = (number of very satisfied cases + number of satisfied cases)/total number of cases × 100%.
4. Functional recovery: Functional recovery was assessed using the Lower Extremity Functional Assessment Scale. The outcomes were categorized into four grades: excellent, good, fair, and poor. Specific evaluation criteria were based on a percentage system, assessing six aspects: joint range of motion, muscle strength, sensory recovery, appearance, complications, and weight-bearing walking ability. The scoring criteria were as follows: Excellent: 100–80 points; Good: 79–60 points; Fair: 59–40 points; Poor: Below 39 points. Functional recovery rate = (Number of excellent cases + Number of good cases)/Total number of cases × 100%.
5. Quality of life: One year postoperatively, the 36-Item Short Form Survey Instrument (SF-36) was used to assess the patient's quality of life (11), including eight dimensions: Bodily Pain (BP), Physical Functioning (PF), Role Limitations due to Physical Health (RP), General Health Perceptions (GH), Social Functioning (SF), Role Limitations due to Emotional Problems (RE), Mental Health (MH), and Vitality (VT). Each dimension was scored on a scale of 0–100, with higher scores indicating better quality of life.

### Statistical analysis

Data statistical analysis and graphing were performed using GraphPad Prism 9.5.0 software (GraphPad Software Inc., San Diego, CA, USA). The Shapiro–Wilk test was used to assess normality of distribution. Continuous variables that met the criteria for normal distribution were expressed as mean ± standard deviation, and comparisons between groups were performed using the independent samples *t*-test. Categorical variables were expressed as counts and percentages, and comparisons between groups were performed using the chi-square test. *P* values were calculated using two-sided tests, and *P* < 0.05 was considered statistically significant.

## Results

### Comparison of baseline characteristics between the two groups

A total of 90 patients with lower limb bone exposure wounds were included in this study. They were divided into two groups based on the type of flap repair: the local flap group (*n* = 50) and the free flap group (*n* = 40). There were no statistically significant differences between the two groups in terms of general characteristics such as gender, age, BMI, location of bone exposure, and causes of injury (all *P* > 0.05), as shown in Table 1. The results indicate that the two groups are highly comparable.

**Table 1:** Comparison of baseline data between the two groups of patients.

Variable	Local flap (*n* = 50)	Free flap (*n* = 40)	*t*/*χ*2	*P*
Age (years)	38.48 ± 3.85	39.10 ± 4.06	0.741	0.461
Gender (M/F)				
M	28 (56.00%)	25 (62.50%)	0.388	0.534
F	22 (44.00%)	15 (37.50%)		
BMI (kg/m^2^)	22.54 ± 0.55	22.74 ± 0.63	1.627	0.107
Location of bone exposure (*n*, %)				
Proximal tibia	12 (24.00%)	9 (22.50%)	0.040	0.998
Middle tibia	15 (30.00%)	12 (30.00%)		
Proximal tibia	10 (20.00%)	8 (20.00%)		
Fibula	13 (26.00%)	11 (27.50%)		
Causes of injury (n, %)				
Open fracture	30 (60.00%)	20 (50.00%)	1.373	0.712
Infection	8 (16.00%)	6 (15.00%)		
Post-traumatic necrosis	10 (20.00%)	12 (30.00%)		
Others	2 (4.00%)	2 (5.00%)		

BMI, Body mass index. Causes of injury classified as “Others” included crush injuries (*n* = 2 in local flap group; *n* = 1 in free flap group) and electrical burns (*n* = 0 in local flap group; *n* = 1 in free flap group).

### Comparison of surgical-related indicators between the two groups of patients

We compared surgical-related indicators (surgical time, intraoperative blood loss, and hospital stay) between the two groups of patients. The results showed that compared with the local flap group, the free flap group had longer surgical duration [(150.6 ± 8.589) min vs. (163.7 ± 7.710) min, *t* = 7.568, *P* < 0.001] and greater intraoperative blood loss [[(150.8 ± 10.50) ml vs. (181.1 ± 12.64) ml, *t* = 12.420, *P* < 0.001], but shorter hospital stays [(18.72 ± 3.534) days vs. (14.23 ± 2.904) days, *t* = 6.480, *P* < 0.001], with statistically significant differences (*P* < 0.05). These results suggest that free flap transplantation surgery is more traumatic and technically more complex, but patients recover and are discharged from the hospital faster postoperatively.

### Comparison of complication rates between the two groups

**Figure 1:**
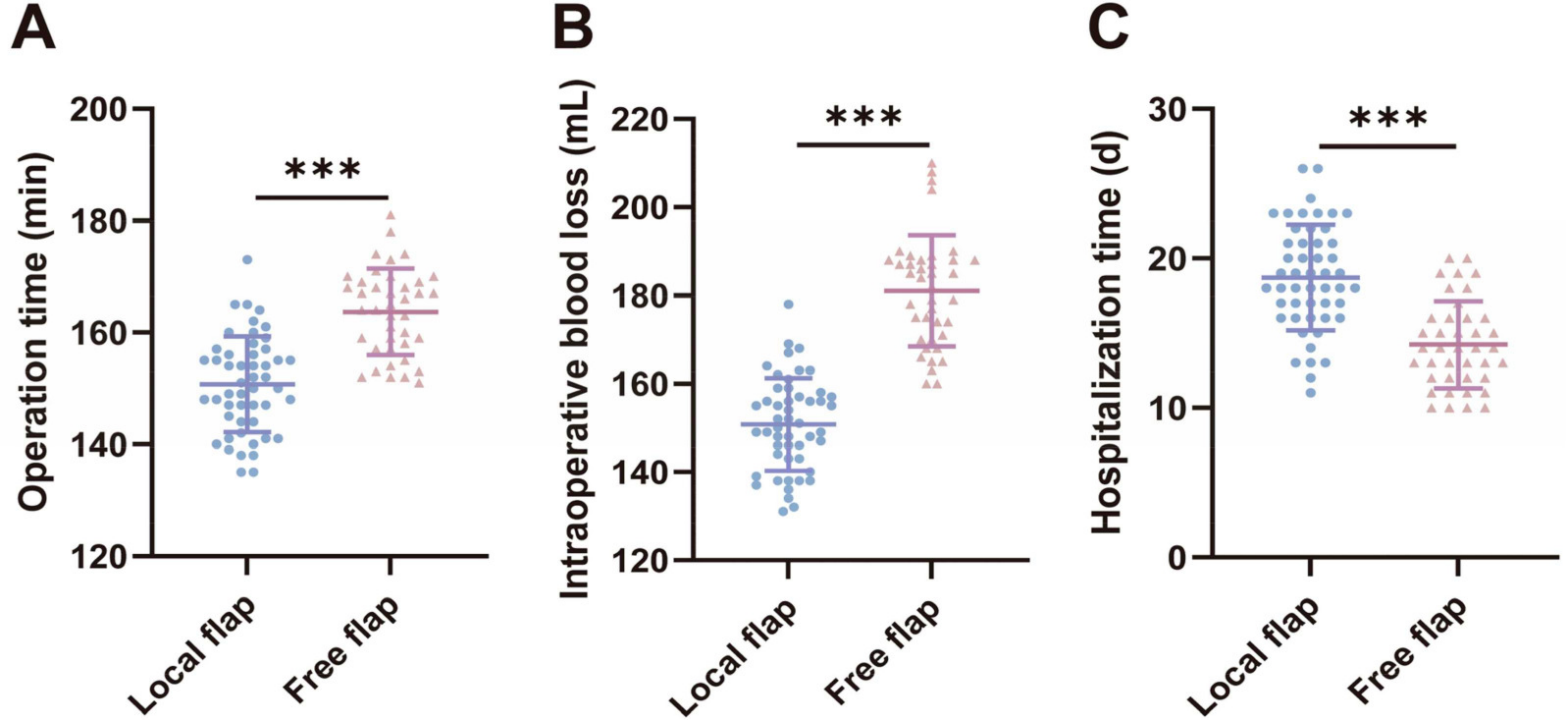
Comparison of surgery-related indicators between the two groups of patients.

Analysis of complication incidence in the two groups revealed that, for individual complications, the rates of flap necrosis (7.50% vs. 2.00%), infection (5.00% vs. 2.00%), vascular crisis (5.00% vs. 0.00%), scar contracture (7.50% vs. 4.00%), and osteomyelitis (5.00% vs. 2.00%) were higher in the free flap group than in the local flap group, but these differences were not statistically significant (all *P* > 0.05) (Table 2). In terms of overall complication rates, the local flap group had a complication rate of 10.00% (5/50), which was significantly lower than the 30.00% (12/40) in the free flap group (*χ*^2^ = 5.802, *P* = 0.016) (Table 2), indicating that local flap transfer offers a superior advantage in controlling overall postoperative complications (Figure 1).

**Table 2:** Comparison of complication rates between the two groups of patients.

Project	Local flap (*n* = 50)	Free flap (*n* = 40)	χ2	*P*
Flap necrosis	1 (2.00%)	3 (7.50%)	0.621	0.431
Infection	1 (2.00%)	2 (5.00%)	0.621	0.431
Vascular crisis	0 (0.00%)	2 (5.00%)	1.264	0.261
Scar contracture	2 (4.00%)	3 (7.50%)	0.519	0.471
Osteomyelitis	1 (2.00%)	2 (5.00%)	0.621	0.431
Overall incidence rate (%)	5 (10.00%)	12 (30.00%)	5.802	0.016

### Comparison of cosmetic satisfaction between the two groups of patients at 1 year postoperatively

The assessment of cosmetic satisfaction at 1 year postoperatively showed (Table 3): the local flap group had a satisfaction rate of 82.00% (41/50), while the free flap group had a satisfaction rate of 92.50% (37/40). The satisfaction rate in the free flap group was slightly higher than that in the local flap group, but the difference was not statistically significant (*χ*^2^ = 2.120, *P* = 0.145). This indicates that there is no significant difference in the long-term aesthetic improvement outcomes between the two flap repair methods.

**Table 3:** Comparison of cosmetic satisfaction between the two groups of patients one year after surgery.

Project	Local flap (*n* = 50)	Free flap (*n* = 40)	*χ* ^2^	*P*
Very satisfied (*n*, %)	18 (36.00%)	20 (50.00%)		
Satisfaction (*n*, %)	23 (46.00%)	17 (42.50%)		
Dissatisfied (*n*, %)	9 (18.00%)	3 (7.50%)		
Appearance satisfied (*n*, %)	41 (82.00%)	37 (92.50%)	2.120	0.145

### Comparison of functional recovery outcomes between the two groups of patients at 1 year postoperatively

Analysis of functional recovery outcomes at 1 year postoperatively (Table 4) showed that the excellent functional recovery rate in the free flap group was 87.50% (35/40), significantly higher than the 70.00% (35/50) in the local flap group, with a statistically significant difference (*χ*^2^ = 3.938, *P* = 0.047). Among these, the proportion of patients in the “excellent” category in the free flap group (47.50%) was significantly higher than that in the local flap group (24.00%) (*χ*^2^ = 5.435, *P* = 0.020). There were no statistically significant differences between the groups for the remaining categories (good, poor, and very poor) (all *P* > 0.05). These results indicate that free flap transplantation is more advantageous in promoting postoperative limb function recovery in patients.

**Table 4:** Comparison of functional recovery between the two groups of patients one year after surgery.

Project	Local flap (*n* = 50)	Free flap (*n* = 40)	*χ* ^2^	*P*
Excellent (*n*, %)	12 (24.00%)	19 (47.50%)	5.435	0.020
Good (*n*, %)	23 (46.00%)	16 (40.00%)	0.326	0.568
Fair (*n*, %)	13 (26.00%)	4 (10.00%)	3.713	0.054
Poor (*n*, %)	2 (4.00%)	1 (2.50%)	0.155	0.694
Functional recovery rate (%)	35 (70.00%)	35 (87.50%)	3.938	0.047

### Quality of life assessment one year after surgery in two groups of patients

**Figure 2:**
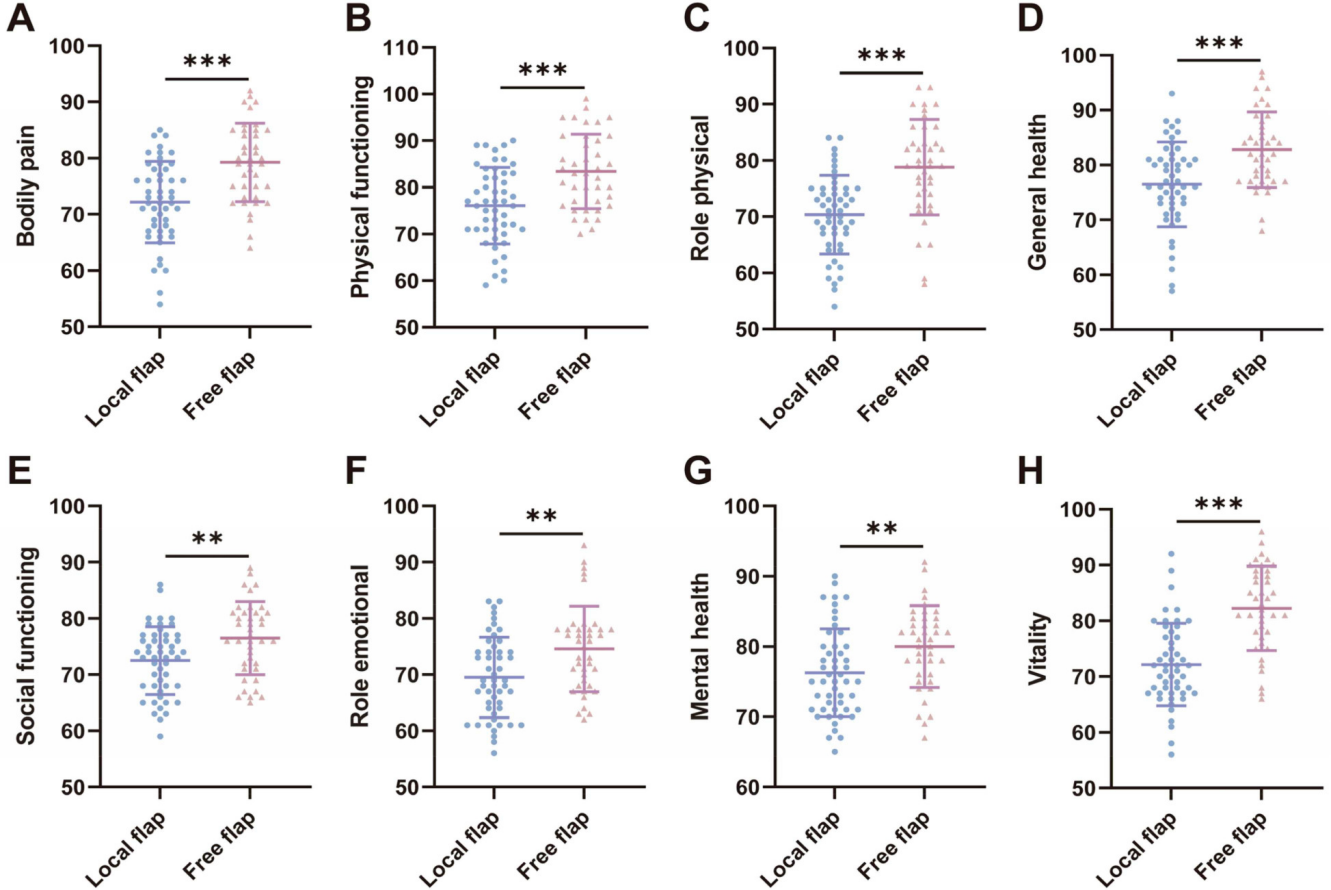
Comparison of quality of life before and after treatment between the two groups of patients.

Additionally, we used the SF-36 to evaluate the quality of life of the two groups of patients one year postoperatively from various dimensions. The results showed (Figure 2) that the free flap group had significantly higher scores than the local flap group in all eight dimensions of the SF-36: Bodily Pain (79.25 ± 6.96 vs. 72.20 ± 7.26, *t* = 4.663, *P* < 0.001), Physical Functioning (83.40 ± 7.98 vs. 76.06 ± 8.19, *t* = 4.273, *P* < 0.001), Role Physical (78.83 ± 8.50 vs. 70.36 ± 7.01, *t* = 5.179, *P* < 0.001), General Health (82.90 ± 6.84 vs. 76.48 ± 7.70, *t* = 4.160, *P* < 0.001), Social Functioning (76.48 ± 6.50 vs. 72.50 ± 6.02, *t* = 3.003, *P* < 0.01), Role Emotional (74.60 ± 7.61 vs. 69.50 ± 7.14, *t* = 3.272, *P* < 0.01), Mental Health (80.00 ± 5.80 vs. 76.26 ± 6.25, *t* = 2.912, *P* < 0.01), and Vitality (82.05 ± 7.59 vs. 72.29 ± 7.38, *t* = 6.225, *P* < 0.001). These results indicate that free flap transplantation has certain advantages in improving patients' overall quality of life.

## Discussion

The repair of lower extremity bone-exposing wounds is one of the important challenges in the field of surgery. As a commonly used and effective treatment, flap reconstruction has been widely applied in clinical practice (1, 12). This study further clarifies the advantages and indications of the two techniques—local flap transfer and free flap transplantation—by comparing their clinical outcomes, thereby providing evidence for clinical decision-making.

Local flap transfer and free flap transplantation differ significantly in their technical principles, which directly leads to different outcomes in surgical-related indicators. Local flap transfer relies on normal skin tissue surrounding the defect, covering the wound through rotation or advancement without the need for vascular anastomosis. Therefore, the surgical procedure is relatively simple, with shorter operating times and less intraoperative bleeding (13, 14). Its advantages include maintaining the continuity of blood supply between the flap and the original tissue, minimal trauma, and lower requirements for microsurgical techniques, making it suitable for implementation in primary healthcare facilities (15, 16). Free flap transplantation requires microsurgical techniques to transplant a vascularized flap from a distant site to the defect area and anastomose vessels to restore blood supply, resulting in higher surgical complexity, longer operative time, and increased bleeding (17, 18). However, this procedure results in shorter postoperative hospital stays, potentially due to more reliable blood supply and more thorough wound healing, thereby reducing the need for prolonged dressing changes and observation (19, 20).

The overall complication rate in the local flap group (10.00%) was significantly lower than that in the free flap group (30.00%), which is closely related to the technical characteristics of the two surgical procedures. Local flaps rely on the blood supply from surrounding tissues, which is primarily derived from the subdermal plexus via the flap base, and they typically follow a random pattern of perfusion. The base of the flap must be handled carefully to avoid stretching or twisting, which could compromise vascular inflow. In general, local flaps should adhere to a length-to-width ratio not exceeding 3:1–4:1 to prevent distal tip ischemia, especially if the base requires rotation during inset. This relatively stable vascular anatomy results in a 0% incidence of postoperative vascular complications and lower risks of flap necrosis and infection. The donor site can often be directly sutured, further reducing the risk of wound exposure and infection (21). Free flap transplantation requires high-level vascular anastomosis techniques, resulting in a higher incidence of postoperative vascular spasm, embolism, and other complications, with a slightly higher risk of flap necrosis (7.50%) compared to local flaps (2.00%). Additionally, the donor site is taken from a distant location, resulting in greater trauma and potentially increasing the risk of complications such as scar contracture (22). This suggests that free flap surgery should be performed by an experienced microsurgical team, with enhanced postoperative vascular monitoring (e.g., skin temperature, capillary refill monitoring) to reduce risks.

In terms of aesthetic satisfaction, the free flap group (92.50%) was slightly higher than the local flap group (82.00%), but the difference was not statistically significant. This may be because free flaps can be precisely tailored to the shape of the defect, and the donor site selection range is broader, making it easier to match the recipient site's skin color and texture (23); in contrast, local flaps may exhibit tension-induced bulging after transfer due to restrictions from adjacent tissues, primarily manifested as high flap tension and impaired venous return (24, 25), which may explain the slightly lower satisfaction rate. In terms of functional recovery, the excellent rate (87.50%) in the free flap group was significantly higher than that in the local flap group (70.00%), with a particularly significant difference in the proportion of “excellent” ratings (47.50% vs. 24.00%). This is attributed to the rich blood supply and strong infection-resistant capabilities of free flaps, which can better cover deep tissues (such as tendons and bones), providing a stable soft tissue bed for functional reconstruction (26, 27). For example, the anterolateral thigh flap can carry muscle tissue, aiding in the repair of cases with concomitant muscle defects and improving limb motor function (28, 29).

Free flap groups scored significantly higher on all eight dimensions of the SF-36 scale, with particularly notable advantages in physical pain and physical function. This may be related to the following factors: free flaps provide more thorough coverage, reducing chronic neuropathic pain caused by prolonged non-healing of the wound (30, 31); improvements in physical and social functioning are related to the restoration of limb mobility (32), such as the ability to walk or bear weight after lower extremity reconstruction, thereby enabling patients to return to work and social activities (33, 34); although there was no statistically significant difference in aesthetic improvement, patients may have more positive subjective perceptions, which may alleviate anxiety and enhance mental health and social functioning (35). The significant improvement in quality of life observed in the free flap group underscores its value beyond mere wound closure. For patients with high functional demands or those for whom long-term quality of life is a primary concern, the benefits of free flap transplantation—despite its higher technical complexity and complication risk—may justify its selection. This finding highlights the importance of incorporating patient-reported outcome measures (PROMs), such as the SF-36, into the preoperative counseling and shared decision-making process. It allows surgeons and patients to weigh the short-term risks against the potential long-term gains in overall well-being, facilitating a more personalized treatment approach.

Our findings are consistent with previous studies comparing local and free flaps for lower extremity reconstruction. For instance, Bhullar et al. reported that free flaps were associated with better patient-reported outcomes in complex lower limb fractures, despite higher complication rates, which aligns with our results showing superior functional recovery and quality of life in the free flap group (36). While we used the SF-36, a generic health status instrument, future studies could consider incorporating limb-specific patient-reported outcome measures (PROMs) such as the LIMB-Q to capture more targeted functional and aesthetic outcomes (37). Additionally, in this study, scar contracture was defined clinically as any degree of contracture that impaired limb mobility; however, the use of objective measurements, such as a goniometer, would provide more precise data in future assessments.

Based on the results of this study and clinical practice, the selection of the two surgical approaches should follow these principles: Local flap transfer is suitable for patients with good wound bed conditions and sufficient adjacent tissue. Its advantages of less trauma and fewer complications make it more appropriate for elderly patients, those with multiple comorbidities, or individuals with low tolerance for surgical risks. The decision for a local flap often hinges on the feasibility of mobilizing adjacent tissue without excessive tension and the absence of significant contamination or infection in the recipient bed. Free flap transplantation is better suited for the repair of complex wounds, especially in functionally important areas (such as around joints) or in younger patients with higher demands for functional recovery. Key considerations for choosing a free flap include the need for well-vascularized tissue to cover exposed vital structures (e.g., bone, tendon), the availability of suitable recipient vessels outside the zone of injury, and the patient's overall physiological reserve to withstand a longer and more complex procedure. Although the risks are higher, it significantly improves long-term quality of life. In addition, patient-specific factors should also be considered, such as vascular condition (local flaps are preferred for diabetic patients), skin elasticity at the donor site, and postoperative rehabilitation compliance, to achieve personalized treatment. This study's cohorts consisted of wounds of comparable size; however, the inherent selection bias in choosing the flap type based on individual patient and wound characteristics (e.g., depth of defect, presence of concomitant tendon/muscle injury, surgeon expertise) remains a limitation of this retrospective analysis.

This study is a single-center retrospective analysis with a limited sample size and potential selection bias; the follow-up period was only one year, and long-term outcomes (such as flap contracture and functional deterioration) require further observation. Additionally, economic factors such as detailed cost comparisons between the two techniques (including operative time, resource utilization, and long-term care costs) were not evaluated and represent an important area for future health economic analyses. Future studies could include multicenter prospective research comparing the efficacy of different types of flaps (e.g., perforator flaps vs. traditional free flaps), incorporating cost-effectiveness analyses, and incorporating three-dimensional reconstruction techniques to optimize flap design, thereby further enhancing repair outcomes.

In summary, local flap transfer offers the advantages of smaller surgical trauma and fewer complications. Although free flap transplantation is more complex and carries a higher risk of complications, it offers superior outcomes in terms of functional recovery and improvement in quality of life. Clinically, the choice should be made based on wound characteristics, patient needs, and medical conditions to precisely select the most appropriate approach and maximize patient benefits.

## Data Availability

The original contributions presented in the study are included in the article/Supplementary Material, further inquiries can be directed to the corresponding author.
